# A Simplified Three-Item Clinical Score to Identify Exertional Hypoxemia in Fibrotic Interstitial Lung Disease: A Real-World Cohort Study

**DOI:** 10.3390/jcm14217858

**Published:** 2025-11-05

**Authors:** Rogerio Rufino, Isabela Tamiozzo Serpa, Leonardo Palermo, Elizabeth Bessa, Bruno Rangel, Mariana Lopes, Agnaldo José Lopes, Mariana Costa Rufino, Cláudia Henrique da Costa, Anamelia Costa Faria

**Affiliations:** 1 Department of Thoracic Diseases, Faculty of Medical Sciences, Rio de Janeiro State University, Rio de Janeiro 20551-030, Brazil; belatamiozzo@gmail.com (I.T.S.); leopbruno@gmail.com (L.P.); elizabethjcb10@yahoo.com.br (E.B.); brunocmhfa@gmail.com (B.R.); marianacarneirolopes@gmail.com (M.L.); agnaldolopes.uerj@gmail.com (A.J.L.); ccosta.uerj@gmail.com (C.H.d.C.); costafaria@gmail.com (A.C.F.); 2 Faculty of Medicine, São Paulo University, São Paulo 01246-903, Brazil; rufinocmariana@gmail.com

**Keywords:** interstitial lung disease, fibrotic interstitial lung disease, oxygen desaturation, real-world study, clinical prediction score

## Abstract

**Background:** Exertional oxygen desaturation (SpO_2_ ≤ 88%) during the six-minute walk test (6MWT) is a key prognostic marker in interstitial lung disease (ILD), yet access to the test is often limited in clinical practice. Developing simple, bedside tools to identify patients at risk may support early risk stratification and guide clinical decision-making. **Methods:** We conducted a retrospective, real-world cohort study in a tertiary referral center between January 2024 and July 2025, including 150 patients, of whom 67.33% (101 patients) were using supplemental oxygen. Clinical and physiological data collected within 30 days of the 6MWT were analyzed. The primary outcome was exertional hypoxemia, defined as peripheral oxygen saturation (SpO_2_) ≤ 88% at the end of the test. Four predictive approaches were evaluated: multivariable logistic regression, stepwise logistic regression, and a simplified clinical score (0–3). The simplified score assigned one point for each of the following: forced vital capacity (FVC) ≤ 61% predicted, diffusing capacity for carbon monoxide (DLCO) ≤ 53% predicted, and presence of chronic cough. Model performance was assessed by receiver operating characteristic (ROC) curves, sensitivity, specificity, predictive values, and risk stratification. **Results:** The simplified score demonstrated robust discriminative performance, comparable to more complex statistical models, with high sensitivity and acceptable specificity. A threshold of ≥2.0 points identified patients at high risk for exertional desaturation with 100% sensitivity and 0.66 specificity. Observed desaturation risk increased progressively across score categories: 17.1% for scores 0–1 (low risk), 58.6% for score 2 (intermediate risk), and 95.1% for score 3 (high risk). **Conclusions:** Compared with multivariable models, the simplified 0–3 clinical score—based on widely available variables (FVC ≤ 61%, DLCO ≤ 53%, and chronic cough)—maintained similar predictive performance (AUC 0.82) with greater operational simplicity. Owing to its high sensitivity and bedside applicability, it represents a promising screening tool for identifying patients at high risk of exertional desaturation, particularly when the 6MWT is unavailable.

## 1. Introduction

Oxygen therapy is a cornerstone intervention in chronic lung diseases, classically indicated for patients with severe hypoxemia—defined as a partial pressure of arterial oxygen (PaO_2_) ≤ 55 mmHg or peripheral oxygen saturation (SpO_2_) ≤ 88% at rest—or for those with intermediate oxygen levels (PaO_2_ 55–59 mmHg) in the presence of cor pulmonale or polycythemia [1]. These indications stem from landmark clinical trials in the 1970s conducted in patients with chronic obstructive pulmonary disease (COPD), in which ≥ 15 h per day of long-term oxygen therapy (LTOT) significantly reduced mortality [2]. Since then, oxygen therapy has also been recommended based on the same criteria for interstitial lung diseases (ILDs), although the supporting evidence is less robust and often extrapolated from COPD populations [3].

Among ILDs, idiopathic pulmonary fibrosis (IPF) is the most frequent phenotype and carries the worst prognosis, with a median survival of only 2–3 years after diagnosis [4]. More recently, the concept of progressive pulmonary fibrosis (PPF) has been introduced to encompass heterogeneous ILD subtypes that share a similarly relentless clinical trajectory [5]. In this setting, hypoxemia may be absent at rest but frequently manifests during exertion. Desaturation during the 6 min walk test (6MWT) is a reproducible marker of poor prognosis and an independent predictor of mortality in fibrotic ILD, and it is a primary physiological criterion for prescribing oxygen [6,7]. Despite its widespread use, clinical trials have yielded mixed results: oxygen supplementation can improve dyspnea and exercise tolerance, yet consistent survival benefits remain elusive [1,8]. Current international guidelines recommend LTOT for severe resting hypoxemia and ambulatory oxygen for exertional desaturation, but these recommendations are based on low-certainty evidence [9,10].

Persistent logistical barriers further complicate optimal oxygen therapy decisions. Access to specialized centers remains limited in many regions, and standardized functional assessments, including the 6MWT and polysomnography, are not universally available [1,11]. Moreover, the need for trained personnel, appropriate facilities, and reproducible testing protocols often restricts the use of 6MWT to tertiary care settings. As a result, many patients with exertional hypoxemia may go unrecognized or remain undertreated [1,12].

Given these challenges, there is a clear clinical need for simple, pragmatic tools capable of identifying ILD patients at risk of exertional hypoxemia, particularly in environments where standardized exercise testing is unavailable [13]. In this study, we aimed to address this gap by developing and evaluating predictive approaches that integrate easily obtainable clinical information—notably the presence of cough—with routine pulmonary function measures (FVC and DLCO). Our objective was to assess whether a simplified clinical–functional score could accurately predict exertional hypoxemia, defined as SpO_2_ ≤ 88% at the end of the 6MWT, thereby informing day-to-day oxygen therapy decisions and improving patient triage.

## 2. Methods

### 2.1. Study Design

This was a retrospective, observational, real-world study based on electronic medical record review of patients with fibrotic interstitial lung disease followed at Piquet Carneiro University Polyclinic between January 2024 and July 2025. The protocol was approved by the Institutional Research Ethics Committee (CAAE: 80166424.3.0000.5259) and conducted in accordance with national and international ethical standards for human research (Supplementary Table S1).

### 2.2. Population and Inclusion Criteria

Patients with a confirmed diagnosis of idiopathic pulmonary fibrosis (IPF) or progressive pulmonary fibrosis (PPF), established according to the clinical, radiological, and physiological criteria in force at the time of care, were included in the study [5,14]. To ensure comparability across variables, only cases with ancillary tests performed within a maximum interval of 30 days were considered eligible. Patients with missing values for FVC, DLCO, or cough status were excluded (n = 12); their demographic and clinical characteristics were comparable to those of the analyzed cohort.

### 2.3. Sampling

The study was primarily designed to develop and internally validate a simple clinical–functional score for predicting exertional hypoxemia, rather than to detect small between-group differences. Based on conventional statistical assumptions (α = 0.05, β = 0.20, power = 80%) and a planned 2:1 allocation of home oxygen users to non-users, a total sample size of approximately 120–150 participants was considered sufficient to ensure an adequate number of events per variable for multivariable modeling, reliable estimation of classification metrics, and internal validation.

### 2.4. Data Sources

Data were extracted from electronic medical records and included:

- Clinical characteristics: age, sex, presence of cough, and smoking history;
- Physiological parameters: forced vital capacity (FVC% predicted) and diffusing capacity for carbon monoxide (DLCO% predicted);
- Oxygenation: peripheral oxygen saturation (SpO_2_) at rest and during exertion;

Pulmonary function tests (spirometry and DLCO) and the 6MWT were performed and interpreted in accordance with ATS/ERS consensus guidelines and standard operating procedures of the local laboratory [15,16,17]. Patients receiving long-term oxygen therapy performed the 6MWT while using their prescribed oxygen flow.

### 2.5. Outcome

Primary outcome. Exertional hypoxemia defined as SpO_2_ ≤ 88% at the end of the 6 min walk test (6MWT), performed and interpreted per ATS/ERS standards, with equipment maintenance/calibration per manufacturer and society recommendations and standardized corridor length/encouragement as specified by ATS/ERS guidance [17,18]. SpO_2_ values were recorded at test completion, as nadir measurements were not consistently documented in the medical records. Secondary outcomes. (i) Home oxygen use at the index visit (baseline characteristic), and (ii) exploratory resting hypoxemia (SpO_2_ ≤ 88% at rest), when available [1,19]. Secondary outcomes were not used to train or select the primary predictive models. All models were developed to discriminate the primary outcome.

Cough was considered present when patient-reported symptoms persisted continuously for ≥8 weeks [20].

### 2.6. Statistical Analysis

Discriminative analyses used SpO_2_ ≤ 88% during the 6 min walk test (6MWT) as the reference outcome for receiver operating characteristic (ROC) analysis, including area under the curve (AUC), sensitivity, specificity, accuracy, positive predictive value (PPV), and negative predictive value (NPV), all at the Youden-optimal threshold. Comparisons stratified by oxygen-use status were considered baseline descriptive analyses and were conducted separately, with patients categorized as home-oxygen users or non-users. Continuous variables were summarized as mean ± standard deviation (SD) or median [interquartile range, IQR], according to distribution. Normality was assessed using the Shapiro–Wilk test and homoscedasticity with Levene’s test. Between-group comparisons employed Student’s *t* test (or Welch’s *t* test when variances were unequal) under normality assumptions, and the Mann–Whitney U test otherwise. Categorical variables were reported as counts (percentages) and compared using Pearson’s χ^2^ test; when any expected cell frequency was <5, the Fisher exact test was applied. For multi-category comparisons (e.g., diagnostic groups), Cramér’s V was calculated as an effect-size measure. Confidence intervals for AUC were estimated using 1000 bootstrap replicates. All statistical tests were two-sided with α = 0.05. Analyses were performed in a validated statistical environment, and ROC analyses, descriptive statistics, and hypothesis tests were conducted using GraphPad Prism v10.6. Discriminative analyses for exertional hypoxemia used SpO_2_ ≤ 88% during the 6 min walk test (6MWT) as the reference standard [21]:

1. Univariable ROC analyses (reference markers): ROC curves for FVC% predicted and DLCO% predicted were generated, reporting AUC and Youden-optimal cut-offs. Shapiro–Wilk results guided descriptive summaries (mean ± SD vs. median [IQR]);
2. Multivariable logistic regression model: A conventional logistic model was constructed including FVC% predicted, DLCO% predicted, and cough as independent predictors. Regression coefficients (β) were estimated from the cohort data, and the linear predictor was used for discrimination and performance metrics.
  Equation—Logit(p) = β0 + β1·FVC + β2·DLCO + β3·Cough
3. Stepwise logistic regression model: A stepwise selection procedure (bidirectional, based on Akaike information criterion) was applied to identify the most informative subset of predictors among the same candidate variables. Final model coefficients were derived from this data-driven selection.
  Equation—Logit(p) = β0 + β1·FVC + β2·DLCO
4. Simplified clinical score (0–3): A point-based score was created by assigning 1 point each for FVC% predicted ≤ 61, DLCO% predicted ≤ 53, and presence of cough (total range 0–3 points). Cut-offs for FVC ≤ 61% and DLCO ≤ 53% were derived from the present cohort using Youden’s index from univariate ROC curves. This pragmatic scoring system was designed for bedside triage, with ≥2 points prespecified as the threshold for high risk.
  Equation—Score = (FVC ≤ 61) + (DLCO ≤ 53) + (Cough present)

## 3. Results

A total of 150 patients were included; 101 (67.3%) were on home oxygen at baseline and 49 (32.7%) were not. Sex distribution was comparable across groups, with no male predominance. Ethnicity was also similar (*p* = 0.799). Chronic cough was more frequent among oxygen users (77.2% vs. 55.1%; *p* = 0.006), and ever-smoker status was likewise more prevalent (58.4% vs. 30.6%; *p* = 0.001). Age was comparable (65.0 [56.0–72.0] years in oxygen users vs. 65.7 ± 11.5 years in non-users; *p* = 0.439) (Table 1).

In contrast, oxygen users showed more impaired lung function, with FVC 50.8 ± 14.9% vs. 66.1 ± 16.9% (*p* < 0.001) and DLCO 37.0 [25.0–52.0]% vs. 55.0 ± 18.7% (*p* < 0.001). The cut-off values for forced vital capacity (FVC ≤ 61%) and diffusing capacity of the lung for carbon monoxide (DLCO ≤ 53%) were derived from the present dataset, based on the Youden index obtained from receiver operating characteristic (ROC) curve analysis. Resting SpO_2_ was lower in the oxygen group (95.0 [92.8–97.0]%) than in non-users (97.0 [96.0–98.0]%; Mann–Whitney, *p* < 0.001). At the end of the 6 min walk test (6MWT), SpO_2_ also remained lower among oxygen users (84.0 [79.0–87.0]%) compared with non-users (92.1 ± 3.3%; Mann–Whitney, *p* < 0.001) (Table 1).

By diagnosis, in the oxygen group (n = 101) the distribution was HP (hypersensitivity pneumonitis) 32.7% (33/101), CTD-ILD (grouping SSc, RA, MCTD, IPAF) 39.6% (40/101), IPF 24.7% (25/101), and Other 3.0% (3/101); in the non-oxygen group (n = 49), CTD-ILD 49.0% (24/49), IPF 28.6% (14/49), HP 20.4% (10/49), and other 2.0% (1/49). The chi-square test showed no statistically significant difference between distributions (χ^2^(3) = 6.31; *p* = 0.097; Cramér’s V = 0.205), indicating a trend toward higher HP among oxygen users and more other diagnoses among non-users, without statistical significance.

Model performance for the primary outcome (SpO_2_ ≤ 88% during the 6 min walk test [6MWT]) is summarized as follows. Five predictive and statistical approaches were evaluated: univariable ROC analyses for FVC% predicted and DLCO% predicted, multivariable logistic regression, stepwise/best-subset logistic regression, an additive rule, and a simplified clinical–functional score (Table 2).

**Figure 1:**
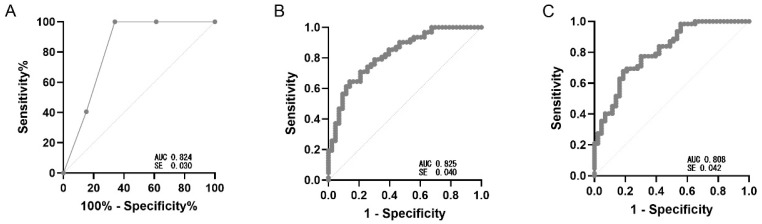
Receiver operating characteristic (ROC) curve for three predictive models of exertional hypoxemia (SpO_2_ ≤ 88%) during the 6 min walk test. Legends: (**A**) Simplified clinical score (0–3), constructed by assigning 1 point each for FVC ≤ 61%, DLCO ≤ 53%, and presence of cough (AUC = 0.824, SE = 0.030). (**B**) Multivariable logistic regression model including FVC, DLCO, and cough as independent predictors (AUC = 0.825, SE = 0.040). (**C**) Stepwise logistic regression model derived using bidirectional selection based on the Akaike information criterion (AUC = 0.808, SE = 0.042). All curves were computed on the same sample.

Discrimination was summarized by AUC with 95% CIs (bootstrap); sensitivity and specificity are reported with 95% CIs. Because PPV/NPV and accuracy vary with prevalence, values shown in Table 2 and Figure 1 reflect the study sample. Stepwise variable selection did not change the model composition, resulting in the same predictors (FVC% predicted, DLCO% predicted, cough) as the multivariable logistic regression. Therefore, only one logistic model is presented. Observed desaturation risk was 17.1% (0–1 points), 58.6% (2 points), and 95.1% (3 points), supporting a three-tier risk stratification (low, intermediate, high) to guide testing priorities (Table 3).

## 4. Discussion

This study presents consistent findings derived from a real-world clinical setting, using clinical and physiological information that is routinely available in the follow-up of patients with fibrotic interstitial lung disease (ILD). The methodological choice of restricting ancillary tests to a maximum interval of 30 days increased the uniformity of measurements and reduced bias related to temporal variation in disease status. In addition, selecting simple variables such as cough, FVC, and DLCO provided not only solid discriminatory performance but also high feasibility for clinical implementation. Clear inclusion criteria and the use of widely accepted statistical methods enhance the reproducibility of these results and strengthen their potential for external validation. The empirical thresholds (FVC ≤ 61% and DLCO ≤ 53%) correspond closely to ranges typically used to denote moderate-to-severe physiological impairment in ILD, supporting their face validity and potential generalizability.

FVC and DLCO remain fundamental parameters for the management and risk stratification of pulmonary fibrosis. A decline of absolute values ≥ 10% in FVC% predicted and ≥15% in DLCO% predicted is associated with poorer outcomes and reflects distinct dimensions of disease severity—volumetric and diffusing capacity impairment, respectively [22,23,24]. Their combined assessment improves prognostic accuracy, particularly in complex scenarios such as combined pulmonary fibrosis and emphysema [25]. In the present study, both parameters were key predictors of exertional desaturation during the 6MWT.

Chronic cough is highly prevalent in fibrotic ILD and is associated with poorer quality of life, higher morbidity, and increased mortality [26]. Incorporating this clinical variable into the predictive model improved its discriminative power, demonstrating that easily obtainable clinical information can meaningfully complement functional measurements for risk stratification.

The 6MWT remains the reference standard for evaluating exertional desaturation and guiding oxygen therapy in patients with ILD and is strongly endorsed by international guidelines [27]. However, its implementation requires strict standardization, appropriate staff training, and adequate physical space. Factors such as musculoskeletal pain, peripheral functional limitations, and variability in testing protocols can influence performance and reproducibility [17,28]. In this context, simple predictive tools become particularly relevant, as they allow initial screening in settings with limited resources or when performing a standard 6MWT is not feasible [29,30].

Recent studies have explored clinical-functional models to anticipate the occurrence of exertional hypoxemia. A previous study reported a C-index of 0.70 for predicting exertional hypoxemia by combining variables such as age, body mass index, diagnosis of idiopathic pulmonary fibrosis, FVC% predicted, and DLCO% predicted [31]. Other analyses demonstrated that most desaturation events occur within the first three minutes of the 6MWT, suggesting the feasibility of abbreviated protocols [32,33]. Our findings are consistent with this evidence, showing that a three-item score, using cough, FVC% predicted, and DLCO% predicted, with greater simplicity and strong potential for clinical implementation. Although discrimination was moderate rather than excellent, the simplicity and bedside feasibility of the score support its clinical adoption as a screening tool.

The proposed scoring system provides a practical framework for triaging patients according to their risk of exertional desaturation. Individuals scoring 0–1 points can be safely managed with routine follow-up, undergoing a 6MWT when available. Those with 2 points should be prioritized for comprehensive functional testing, while patients with 3 points warrant immediate evaluation with 6MWT and consideration of ambulatory oxygen therapy. This pragmatic approach may help clinicians optimize testing resources and identify high-risk individuals earlier, particularly in healthcare settings where access to exercise testing is limited.

Because the score was derived and internally tested within a single cohort, external validation in independent populations is essential before routine clinical adoption. We also acknowledge limitations related to the retrospective design, possible selection bias, and moderate sample size, as well as dependence on internally derived cut-offs. This study has limitations inherent to its retrospective, electronic medical record–based design, including potential selection and information biases, variability in clinical documentation, and incomplete standardization of testing procedures. The relatively modest sample size and the imbalanced distribution between groups (2:1) may limit precision and generalizability and influence prevalence-dependent metrics such as positive predictive value. Nevertheless, the use of widely available variables supports the reproducibility of the proposed score across diverse clinical settings.

Although exertional desaturation prediction has been explored previously, our study provides a minimalist, bedside tool that preserves accuracy (AUC 0.82), ensures maximal sensitivity for screening (cut-off > 2 points: sensitivity 100%), and relies on universally available variables, facilitating use in resource-limited settings.

In summary, our results reinforce the feasibility of using a simple clinical–functional score to identify patients with fibrotic ILD at higher risk of exertional hypoxemia. The combination of cough, FVC% predicted, and DLCO% predicted allows effective initial screening and can help prioritize more complex assessments such as the 6MWT. Owing to its simplicity, low cost, and robust discriminatory performance, the proposed tool can be easily incorporated into clinical workflows, particularly in resource-limited environments. Future studies should externally validate this simple score to confirm its utility across diverse ILD populations.

## Figures and Tables

**Table 1:** Baseline characteristics by home oxygen use (fibrotic ILD cohort).

	Home Oxygen Supply (n.101)	No Home Oxygen Supply (n.49)	*p*-Value	Test
Sex: F/M	71 (70.3%):30 (29.7%)	29 (59.2%)/20 (40.8%)	0.176	χ^2^
Race: White/Non-white	37 (36.6%):64 (63.4%)	19 (38.8%)/30 (61.2%)	0.799	χ^2^
Smoking: Ever	59 (58.4%)	15 (30.6%)	0.001	χ^2^
Smoking: Never	42 (41.6%)	34 (69.4%)	0.001	χ^2^
Age (years)	65.0 (56.0–72.0)	65.0 (60.0–75.0)	0.439	Mann–Whitney
Years on oxygen	1.0 (1.0–2.0)	—	—	—
SpO_2_ at rest (%)	95.0 (92.8–97.0)	97.0 (96.0–98.0)	<0.001	Mann–Whitney
SpO_2_ end of 6MWT (%)	84.0 (79.0–87.0)	92.0 (89.8–94.2)	<0.001	Mann–Whitney
FVC (%)	50.8 ± 14.9	66.1 ± 16.9	<0.001	Welch *t*-test
DLCO (%)	37.0 (25.0–52.0)	54.0 (39.0–66.0)	<0.001	Mann–Whitney

Legend: Data are presented as n (%) for categorical variables and as median [IQR] or mean ± SD for continuous variables. Between-group comparisons were performed using Pearson’s χ^2^ test for categorical variables, and Student’s *t* test or Mann–Whitney U test for continuous variables, as appropriate. All *p* values are two-sided with α = 0.05. Abbreviations: O_2_ = oxygen; SpO_2_ = peripheral oxygen saturation; 6MWT = 6 min walk test; FVC = forced vital capacity; DLCO = diffusing capacity of the lung for carbon monoxide; IQR = interquartile range; SD = standard deviation.

**Table 2:** Discriminative performance for SpO_2_ ≤ 88% during the 6MWT.

Model	AUC	Cut-Off	Sensitivity	Specificity	Accuracy	PPV	NPV
Logistic	0.825 (SE 0.040)	≥0.38	0.90	0.46	0.73	0.75	0.70
Stepwise	0.805 (SE 0.042)	≥0.38	0.84	0.55	0.72	0.73	0.70
Simplified clinical score (0–3)	0.824 (SE 0.030)	≥2.0	1.00	0.66	0.72	0.76	0.64

Legend: Discriminative performance of four predictive models for exertional hypoxemia (SpO_2_ ≤ 88% during the 6 min walk test [6MWT]). The area under the ROC curve (AUC) indicates overall model discrimination. “Cut-off” values represent thresholds that maximize sensitivity and specificity based on the Youden index; for the simplified clinical score, an a priori threshold of ≥2 points was applied. Cut-offs are expressed as percent-predicted values for clinical interpretability. Abbreviations: AUC = area under the ROC curve; PPV = positive predictive value; NPV = negative predictive value.

**Table 3:** Simplified clinical–functional score (0–3) and risk of exertional desaturation (SpO_2_ ≤ 88%).

Score (0–3)	n	Events (SpO_2_ ≤ 88%)	Observed Risk (%)	95% CI	Probability Band
0–1	35	6	17.1%	6.6–33.7	Low (0–40%)
2	29	17	58.6%	38.9–76.5	Intermediate (40–70%)
3	41	39	95.1%	83.5–99.4	High (>70%)

Legend: Scoring system: cough present = 1 point; FVC% predicted ≤ 61% = 1 point; DLCO% predicted ≤ 53% = 1 point (total score range: 0–3). The probability band was categorized as low (<40%), intermediate (40–70%), or high (>70%) according to the observed risk in each score group. Values represent the observed proportion of patients who experienced desaturation during the exertional test, along with the 95% confidence intervals calculated for each category.

## Data Availability

The raw data are available as Supplementary Material.

## Supplementary Materials

The following supporting information can be downloaded at: https://www.mdpi.com/article/10.3390/jcm14217858/s1, Table S1. Checklist S1. STROBE Statement checklist for cohort studies.

## Abbreviations

Abbreviation	Full term
6MWT	6 min walk test
ATS	American Thoracic Society
AUC	Area under the curve
BMI	Body mass index
CI	Confidence interval
COPD	Chronic obstructive pulmonary disease
CPFE	Combined pulmonary fibrosis and emphysema
CTD-ILD	Connective tissue disease–associated interstitial lung disease
DLCO	Diffusing capacity for carbon monoxide
EMR	Electronic medical record
ERS	European Respiratory Society
FVC	Forced vital capacity
HR	Heart rate
ILD	Interstitial lung disease
IMC	Body mass index (Índice de massa corporal)
IPF	Idiopathic pulmonary fibrosis
IQR	Interquartile range
LTOT	Long-term oxygen therapy
MCTD	Mixed connective tissue disease
NPV	Negative predictive value
O_2_	Oxygen
PaO_2_	Partial pressure of arterial oxygen
PPF	Progressive pulmonary fibrosis
PPV	Positive predictive value
PFT	Pulmonary function test
RA	Rheumatoid arthritis
ROC	Receiver operating characteristic
SBP	Systolic blood pressure
SD	Standard deviation
SOP	Standard operating procedure
SpO_2_	Peripheral oxygen saturation
SSc	Systemic sclerosis
STROBE	Strengthening the Reporting of Observational Studies in Epidemiology
V	Cramér’s V (effect size)
