# Supplementary material for: A Simplified Three-Item Clinical Score to Identify Exertional Hypoxemia in Fibrotic Interstitial Lung Disease: A Real-World Cohort Study
Source: J Clin Med. 2025 Nov 5;14(21):7858. doi: 10.3390/jcm14217858 (PMC12610787; doi:10.3390/jcm14217858)
Supplement: Supplementary file 1 [file jcm-14-07858-s001.zip › jcm-3941539-supplementary.pdf]

STROBE Statement—Checklist for Cohort Studies

Manuscript: " A Simplified Three-Item Clinical Score to Identify Exertional Hypoxemia in Fibrotic Interstitial Lung Disease: A Real-World Cohort Study"

STROBE item (cohort)	Recommendation	Where addressed in manuscript
Title/Abstract (1a,1b)	Indicate study design in title/abstract; balanced summary of methods and findings.	Title & Abstract
Background/Rationale (2)	Explain scientific background and rationale.	Introduction (first two paragraphs)
Objectives (3)	State specific objectives, including any prespecified hypotheses.	Introduction (last paragraph)
Study Design (4)	Present key elements of the study design early in the paper.	Methods – Study design
Setting (5)	Describe setting, locations, relevant dates (recruitment, data collection).	Methods – Study design & Setting (Policlínica U. Piquet Carneiro; Jan 2024–Jul 2025)
Participants (6a,6b)	Eligibility criteria, sources/methods of selection; methods of follow-up.	Methods – Population & inclusion criteria; Data sources
Variables (7)	Clearly define outcomes, exposures, predictors, confounders, effect modifiers; diagnostic criteria.	Methods – Outcome (SpO_2≤88% at end-6MWT); Clinical variables (cough≥8 w); PFT definitions (FVC%, DLCO%)
Data Sources/Measurement (8)	For each variable, give sources of data and assessment methods; comparability.	Methods – Data sources; PFTs & 6MWT per ATS/ERS SOPs

Bias (9)	Describe any efforts to address potential sources of bias.	Methods – ≤30-day window between tests; Discussion – EMR-based biases & case-mix
Study Size (10)	Explain how the study size was arrived at.	Methods – Real-world cohort (consecutive availability; no formal power calc)
Quantitative Variables (11)	Explain handling of quantitative variables (groupings, cut-offs).	Methods – Modeling (FVC%≤61, DLCO%≤53; cough×1.3)
Statistical Methods (12a–e)	Describe all statistical methods; subgroup/interaction; missing data; loss to follow-up; sensitivity analyses.	Methods – Statistical analysis (ROC/AUC, Youden; Shapiro–Wilk/Levene; χ^2/Fisher; mean imputation; bootstrap CIs)
Participants (Results 13a–c)	Numbers at each stage; reasons for non-participation; flow diagram.	Results – First paragraph (n=150; oxygen users=101; non-users=49). Flow diagram: Supplement S-Figure
Descriptive Data (14a–c)	Participant characteristics; information on exposures & potential confounders; missing data.	Results – Table 1 & text (age, sex, race, smoking, cough, PFTs, SpO_2)
Outcome Data (15)	Report numbers of outcome events/summary measures over time.	Results – 6MWT SpO_2; score strata risks (Table 3)
Main Results (16a–c)	Unadjusted/adjusted estimates, precision (CI); category boundaries; translate relative risk into absolute risk when relevant.	Results – Model performance (AUC, Se/Sp; Table 2); Risk bands (Table 3 with 95% CI)

Other Analyses (17)	Report subgroup analyses, interactions, sensitivity analyses.	Results/Methods – Stepwise vs score; risk gradient
Key Results (18)	Summarize key results with reference to objectives.	Discussion – Opening paragraph
Limitations (19)	Discuss limitations, potential bias/imprecision; direction/magnitude; generalisability.	Discussion – Study limitations (EMR biases, 2:1 case mix, missing/incomplete tests)
Interpretation (20)	Give a cautious overall interpretation considering objectives, limitations, multiplicity, other evidence.	Discussion – Comparative context with literature; pragmatic utility
Generalisability (21)	Discuss external validity of the results.	Discussion – Real-world setting; feasibility in resource-limited clinics
Funding (22)	Give source of funding and role of funders.	Declarations – Funding (FAPERJ); Competing interests
